# Monoassociation with bacterial isolates reveals the role of colonization, community complexity and abundance on locomotor behavior in larval zebrafish

**DOI:** 10.1186/s42523-020-00069-x

**Published:** 2021-01-21

**Authors:** Chelsea A. Weitekamp, Allison Kvasnicka, Scott P. Keely, Nichole E. Brinkman, Xia Meng Howey, Shaza Gaballah, Drake Phelps, Tara Catron, Todd Zurlinden, Emily Wheaton, Tamara Tal

**Affiliations:** 1 Center for Public Health and Environmental Assessment, US EPA, RTP, NC USA; 2 Oak Ridge Institute for Science and Education, RTP, NC USA; 3 Center for Computational Toxicology and Exposure, US EPA, RTP, NC USA; 4 Center for Environmental Measurement and Modeling, US EPA, Cincinnati, OH USA; 5 Bioanalytical Ecotoxicology Department, Helmholtz Centre for Environmental Research - UFZ, Leipzig, Germany; 6 Present Address: Bioanalytical Ecotoxicology Department, Helmholtz Centre for Environmental Research - UFZ, Leipzig, Germany

**Keywords:** Microbiome, Hyperactivity, Monoassociation, Gnotobiotic, Germ-free, Axenic, Zebrafish, Monocolonization

## Abstract

**Background:**

Across taxa, animals with depleted intestinal microbiomes show disrupted behavioral phenotypes. Axenic (i.e., microbe-free) mice, zebrafish, and fruit flies exhibit increased locomotor behavior, or hyperactivity. The mechanism through which bacteria interact with host cells to trigger normal neurobehavioral development in larval zebrafish is not well understood. Here, we monoassociated zebrafish with either one of six different zebrafish-associated bacteria, mixtures of these host-associates, or with an environmental bacterial isolate.

**Results:**

As predicted, the axenic cohort was hyperactive. Monoassociation with three different host-associated bacterial species, as well as with the mixtures, resulted in control-like locomotor behavior. Monoassociation with one host-associate and the environmental isolate resulted in the hyperactive phenotype characteristic of axenic larvae, while monoassociation with two other host-associated bacteria partially blocked this phenotype. Furthermore, we found an inverse relationship between the total concentration of bacteria per larvae and locomotor behavior. Lastly, in the axenic and associated cohorts, but not in the larvae with complex communities, we detected unexpected bacteria, some of which may be present as facultative predators.

**Conclusions:**

These data support a growing body of evidence that individual species of bacteria can have different effects on host behavior, potentially related to their success at intestinal colonization. Specific to the zebrafish model, our results suggest that differences in the composition of microbes in fish facilities could affect the results of behavioral assays within pharmacological and toxicological studies.

**Supplementary Information:**

The online version contains supplementary material available at 10.1186/s42523-020-00069-x.

## Background

Microbes have co-evolved with their hosts and are required for host health and development, including neurodevelopment and behavior [1]. Across taxa, animals raised under microbe-free conditions or depleted of their intestinal microbes consistently show alterations in behavior and physiology [1, 2]. For example, both mice and zebrafish lacking microbes show increased locomotor activity, with a critical window in development during which exposure to microbes can result in control-like behavior [3–5]. There are several potential mechanisms through which microbes may influence behavioral development. The microbiota-gut-brain axis allows bidirectional communication via activation of the vagus nerve. Another route of influence is through the direct or indirect production of metabolites that can interact with the immune system or penetrate the blood-brain-barrier and exert direct effects on the developing nervous system [2].

While much progress has been made, the specific mechanisms through which microbes influence brain development and behavior remain unknown. In particular, the role of microbial taxonomic and functional diversity is unclear. For example, monoassociation of axenic mice with *Bifidobacterium infantis* was shown to reduce restraint stress and restore normal behavior [6]. In contrast, only a complex microbiota can restore microglia insufficiency in mice [7], suggesting that effects on microglia are not regulated by bacterial load, but may require either diversity or a suite of particular microbial genes [2]. In some cases, colonization with single bacterial strains, even when closely related, can differentially affect host development [8, 9]. For example, in the fruit fly *Drosophila melanogaster*, monoassociation with *Lactobacillus brevis*, but not *L. plantarum*, restored normal locomotor behavior [9].

In a larval zebrafish model, a powerful system for assessing the function of host-microbe interactions [10, 11], heat-killed bacteria and selected microbe-associated molecular patterns were both insufficient to modify axenic-related hyperactivity [4]. Furthermore, rather than restore normal behavior, monoassociation with either *Aeromonas veronii* or *Vibrio cholerae* resulted in a hypoactive phenotype [4]. In a different study, monoassociation of larval zebrafish with *L. plantarum* resulted in control-like locomotion, but reduced cortisol levels following stress, similar to the microbe-free phenotype [5]. Finally, monoassociation with either *Escherichia coli* or *Bacillus subtilis* at different doses only partially reduced axenic-like hyperactivity [12].

Importantly, differences in behavioral effects arising from monoassociation experiments could be mediated, in part, by bacterial abundance, and yet bacterial load is rarely quantified. To our knowledge, confirmation of bacterial identity by sequencing following monoassociation experiments has yet to be applied in zebrafish. A lack of confirmatory sequencing introduces a degree of experimental uncertainty in behavioral manifestations triggered by waterborne inoculation with specific strains of bacteria.

Here, to gain further insight into the mechanisms through which microbes mediate neurobehavioral development and the role of both microbial diversity and absolute abundance, we conventionalized axenic zebrafish with single or mixed host-associated bacterial isolates, as well as with an environmental bacterial isolate. We conducted whole genome sequencing on host-associated isolates and then used 16S rRNA gene sequencing to confirm genus-level identities at the end of the study. Finally, we used droplet digital PCR to quantify the total concentration of bacteria within each host-associated group. Our data suggest the importance of both bacterial identity and intestinal colonization on neurobehavioral development, and further, highlight the importance of understanding host-microbial interactions in animal models used for genetic and toxicity screens.

## Results

### Individual host-associated isolates differ in the ability to block axenic-related hyperactivity

**Fig. 1:**
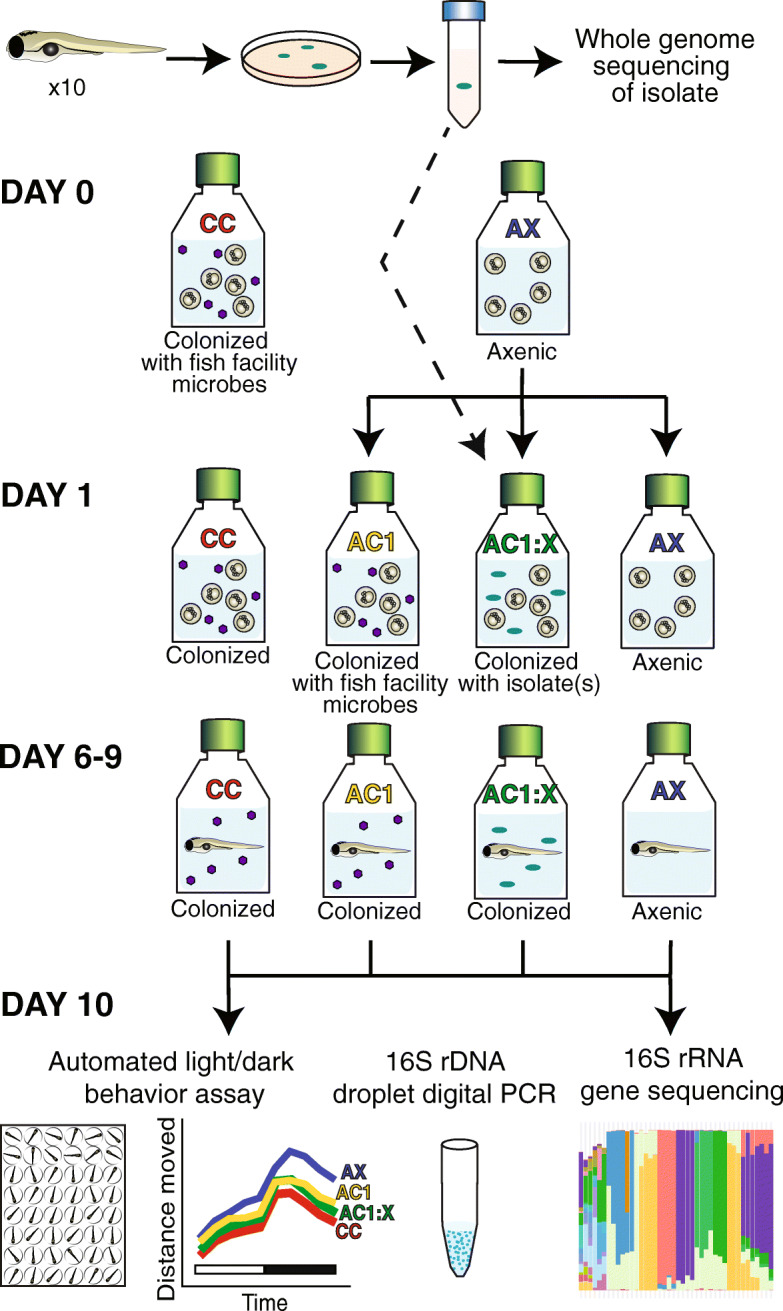
A zebrafish experimental system with different colonization statuses. To generate host-associated bacterial isolates, visually pure isolates were grown from homogenized zebrafish larvae and then underwent whole genome sequencing. To examine the effects of colonization status on behavior, larvae were generated to be conventionally colonized (CC), axenic (AX), or axenic colonized on day 1 with either aquaculture facility microbes (AC1) or isolated single or mixed bacterial species (AC1:X). At 10 days post-fertilization, larvae were assayed for behavior, bacterial identity (i.e.16S rRNA gene sequencing), and bacterial concentration (i.e. 16S rDNA droplet digital PCR). Purple hexagons indicate heterogeneous aquaculture facility microbes. Turquoise ovals indicate strains of microbes isolated from zebrafish used for association experiments

**Fig. 2:**
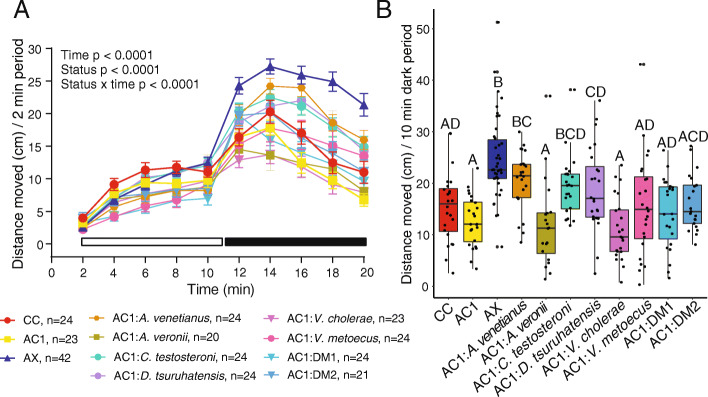
Association with select host-associated bacterial species variably blocked the hyperactive axenic behavioral phenotype (*n* = 4–8 larvae per flask from 3 to 7 flasks per cohort). Distance moved every 2 min in the light and dark periods (**a**; error bars = standard error of the mean) and mean distance moved every 10 min in the dark period (**b**). Different letters indicate significant differences (*p* < 0.05). CC = conventionally colonized; AC1 = axenic colonized on day 1; AX = axenic; AC1:DM1 = *A. venetianus* + *V. metoecus* + *C. testosteroni* + *D. tsuruhatensis + A. veronii*; AC1:DM2 = *A. venetianus* + *C. testosteroni* + *D. tsuruhatensis + A. veronii*

Zebrafish host-associated isolates were obtained from conventionally colonized zebrafish and used for subsequent association experiments to examine the effects of single and mixed isolates on locomotor behavior (Fig. 1). For host-associated single and mixed isolates, there were significant effects from time (*p* < 0.0001), colonization status (*p* < 0.0001), and the interaction between time and status (*p* < 0.0001) on distance moved (Fig. 2a). Further, there was a significant effect of colonization status on mean distance moved during both the light (*p* = 0.00048) and dark (*p* = 5.75 × 10^− 13^) periods. Because light and dark period behavior were correlated (*p* = 3.8 × 10^− 14^, *R*^*2*^ = 0.19) and given our previous observations that the most robust behavioral changes are observed after the light to dark transition [4], we report further behavioral results only for the dark period. As reported previously [4], axenic zebrafish were hyperactive relative to conventionally colonized (CC) or axenic colonized on day 1 (AC1) control groups (Fig. 2b). For host-associated isolates, we found that phenotypically control-like locomotor behavior occurred (i.e., there was no significant difference compared to either CC or AC1 in distance moved) following monoassociation with *Aeromonas veronii, Vibrio cholerae,* or *V. metoecus,* or with either defined mixture (Fig. 2b). In contrast, hyperactivity was only partially blocked by monoassociation with *Comamonas testosteroni* or *Delftia tsuruhatensis* and was not blocked by monoassociation with *Acinetobacter venetianus* (Fig. 2b)*.*

### 16S rRNA gene sequencing confirmed bacterial isolates and revealed potential bacteria in axenic cohort

**Fig. 3:**
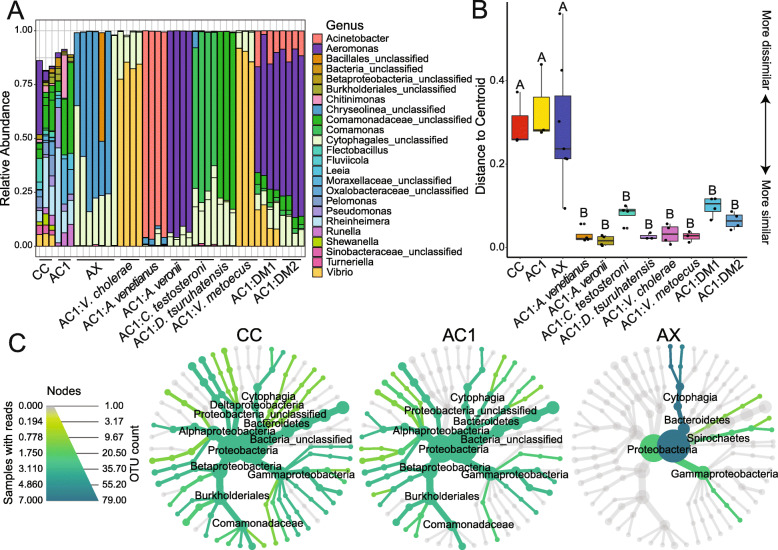
Results of 16S rRNA gene sequencing. Analysis revealed complex bacterial communities in CC and AC1 larvae and verified genus-level identities of conventionalized groups at the end of each experiment (**a**). Distance to centroid on a Bray-Curtis dissimilarity matrix (**b**) revealed high within-group heterogeneity for CC, AC1, and AX, while heterogeneity was low for larvae colonized with isolates. Heat trees (**c**) show differences in microbial diversity between CC, AC1, and AX larvae. CC = conventionally colonized; AC1 = axenic colonized on day 1; AX = axenic; AC1:DM1 = *A. venetianus* + *V. metoecus* + *C. testosteroni* + *D. tsuruhatensis + A. veronii*; AC1:DM2 = *A. venetianus* + *C. testosteroni* + *D. tsuruhatensis + A. veronii*

Using 16S rRNA gene sequencing, we assessed the genus level identity of the bacterial isolates after the 10-day study period. For larvae that were monoassociated, we found that the zebrafish-associated genus matched the inoculant species identity with an average of 85% relative read abundance (Fig. 3a; 95% AC1:*A. venetianus*; 93% AC1:*A. veronii* 70% AC1:*C. testosteroni*; 79% AC1:*D. tsuruhatensis*; 82% AC1:*V. cholerae*; 89% AC1:*V. metoecus*). We further examined within-group differences by calculating the distance to centroid on Bray-Curtis dissimilarity scores (Fig. 3b; *F* = 10.99, *p* = 1.0 × 10^− 4^). There was significantly higher heterogeneity within CC, AC1, and AX compared to all association treatment groups (Fig. 3b). To visualize community differences in diversity between CC, AC1, and AX larvae, we created heat trees. These depict the relative abundance of different OTUs using the color and size of the nodes in a taxonomic tree [13]. A visual comparison of the heat trees shows the complex community composition in CC and AC1, while AX larvae are predominantly colonized by two related genera (Fig. 3c).

Notably, we detected relatively high read counts for two bacterial operational taxonomic units (OTUs; OTU6 and OTU8) in the AX larvae, identified at the order level as Cytophagales (Fig. 3a, c). These OTUs, as well as an additional Cytophagales (OTU7), were also present in axenic larvae colonized with single isolates or defined mixtures but were not detected in CC or AC1 larvae, nor were they present in the negative controls or fish facility water samples (Supplemental data). To examine whether the presence of these three OTUs may have affected zebrafish locomotor behavior in axenic or monoassociated larvae, we used Bayesian ANCOVA with ‘colonization status’ as the fixed effect and included the three OTUs as separate covariates. The best model was ‘colonization status’ alone, with a Bayes factor of 5.5 × 10^5^, which was 1.29 times better than the second-best model ‘colonization status + OTU8’ (Bayes factor of 4.2 × 10^5^). In an additional analysis, including the three OTUs in the null model resulted in a Bayes factor of 27 for ‘colonization status,’ suggesting strong evidence for the alternative hypothesis that the presence of these OTUs did not affect locomotor behavior.

### Bacterial loads differ between groups

**Fig. 4:**
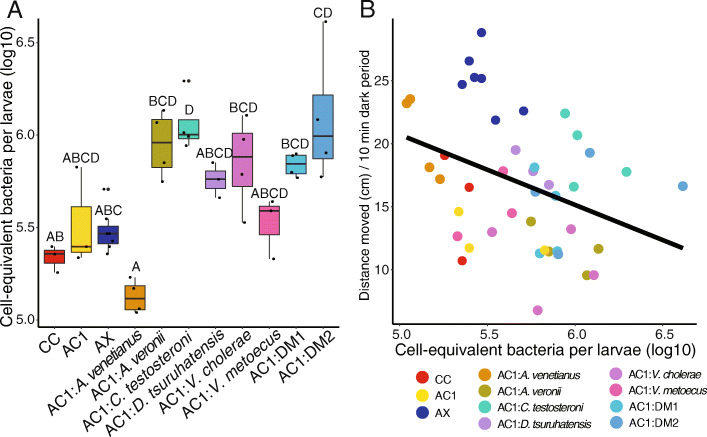
Results of 16S rRNA gene droplet digital PCR. Analysis revealed group differences in bacterial load per larvae (**a**). These differences correlate with behavior (**b**; *p* = 0.017, *R*^*2*^ = 0.11); CC = conventionally colonized; AC1 = axenic colonized on day 1; AX = axenic; AC1:DM1 = *A. venetianus* + *V. metoecus* + *C. testosteroni* + *D. tsuruhatensis + A. veronii*; AC1:DM2 = *A. venetianus* + *C. testosteroni* + *D. tsuruhatensis + A. veronii*

Using 16S rRNA gene ddPCR measurements, we normalized relative abundance to estimate the cell-equivalent total bacterial load per larvae, calibrated with the number of 16S rRNA genes per genome. We found that bacterial load differed significantly between groups (Fig. 4a; *p* = 2.29 × 10^− 4^) and that this difference related to locomotor behavior. The cell-equivalent concentration of bacteria per larvae was weakly but significantly correlated with mean distance moved across all groups (*p* = 0.017, *R*^*2*^ = 0.11; Fig. 4b). Furthermore, accurately measuring bacterial concentrations revealed that the two OTUs (OTU6 and OTU8) detected by 16S rRNA gene sequencing were indeed present at a high concentration in AX larvae, at levels that were not significantly different from most treatment groups (Fig. 4a). Lastly, we examined how well the relative abundance data (i.e., 16S rRNA gene sequencing) reflected the absolute (i.e., ddPCR) bacterial load. Across groups, the log sum of the read counts was positively correlated with the log of the 16S rRNA gene concentration data (*p* = 2.8 × 10^− 10^, *R*^*2*^ = 0.62) (Fig. S1).

### Monoassociation with environmentally-associated isolate does not block axenic-related hyperactivity

**Fig. 5:**
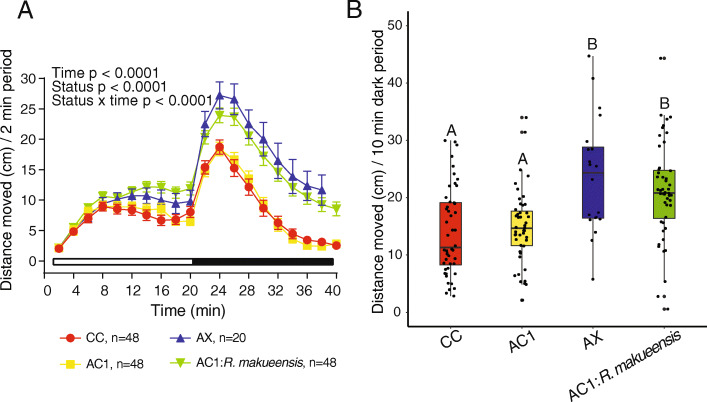
Monoassociation with an environmentally-associated bacteria failed to block axenic-like hyperactivity. Distance moved every 2 min in the light and dark periods (**a**; error bars = standard error of the mean) and mean distance moved every 10 min in the dark period (**b**). CC = conventionally colonized; AC1 = axenic colonized on day 1; AX = axenic

Because we observed variation in the ability of different host-associated bacteria to block hyperactivity, we hypothesized that intestinal colonization may be necessary for neurobehavioral development. To test this, locomotor activity in zebrafish monoassociated with the environmentally-associated isolate *Rheinheimera makueensis* was compared to CC, AC1, and AX larvae. Overall, significant effects of time (*p* < 0.0001), colonization status (p < 0.0001), and the interaction between time and status (p < 0.0001) on distance moved were observed (Fig. 5a). Further, for mean distance moved, there was a significant effect of colonization status (*p* = 8.9 × 10^− 7^), whereby AX larvae and those exposed to *R. makueensis* were hyperactive compared to both CC and AC1 groups (Fig. 5b).

### Isolates that block axenic-related hyperactivity were more abundant than other isolates in defined mixtures

To examine the effect of defined mixtures on bacterial growth patterns, we normalized relative read abundance (i.e., 16S rRNA gene sequencing) by absolute abundance (i.e., 16S rRNA gene ddPCR) for individual species, calibrated with 16S rRNA copies per cell [14]. This resulted in estimates of normalized bacterial loads for each species within both the monoassociation and defined mixture experiments (Table 1). In the monoassociated zebrafish larvae, *A. veronii* had the highest load followed by *C. testosteroni* > *D. tsuruhatensis > V. metoecus* > *A. venetianus*. For larvae associated with Defined Mixture 1, the estimated bacterial loads for each species were lower than the maximum values of the monoassociated zebrafish, particularly for *D. tsuruhatensis* (5.66 vs 4.55) and *C. testosteroni* (5.90 vs 4.15). Ranked by load within Defined Mixture 1, *A. veronii* was the most abundant, followed by *V. metoecus* > *A. venetianus* > *D. tsuruhatensis* > *C. testosteroni*. Notably, the two highest-ranked taxa in this mixture, *A. veronii* and *V. metoecus*, were the species that fully blocked axenic-related hyperactivity (in addition to *V. cholerae*) in monoassociation experiments (Fig. 2b). Compared to Defined Mixture 2, the presence of *V. metoecus* in Defined Mixture 1 was associated with a 0.31 ± 0.05 decrease in the other species (species cell-equivalent log_10_ mean in Defined Mixture 2 – Defined Mixture 1). For Defined Mixture 2, where *V. metoecus* was not added, bacterial loads of *A. veronii* and *A. venetianus* were estimated at comparable loads to those of the monoassociated zebrafish, while *D. tsuruhatensis* and *C. testosteroni* showed significant log reductions, with the ranks as follows: *A. veronii* > *A. venetianus* > *D. tsuruhatensis* > *C. testosteroni* (Table 1). Generation time and growth rate values for monoassociation and mixture experiments are provided in Supplemental Materials.

**Table 1:** Normalized cell-equivalent log_10_ bacterial load values for monoassociation and mixture experiments

Species	Mean ± SD single species	Mean ± SD Defined Mixture 1 (log_**10**_ difference)	Mean ± SD Defined Mixture 2 (log_**10**_ difference)
*A. venetianus*	5.10 ± 0.10	4.75 ± 0.51 (− 0.35)	5.11 ± 0.46 (0.01)
*A. veronii*	5.92 ± 0.19	5.61 ± 0.04 (− 0.31)	5.94 ± 0.33 (0.02)
*C. testosteroni*	5.90 ± 0.18	4.15 ± 0.30 (− 1.75)	4.42 ± 0.39 (− 1.49)
*D. tsuruhatensis*	5.66 ± 0.09	4.55 ± 0.17 (− 1.11)	4.83 ± 0.45 (− 0.82)
*V. cholerae*	5.77 ± 0.24	Not added to mixture	Not added to mixture
*V. metoecus*	5.47 ± 0.16	4.92 ± 0.21 (− 0.55)	Not added to mixture

## Discussion

The interface between host cells and the microbiome is not well understood. While microbe-free animals consistently show altered behavioral phenotypes, including hyperactivity, it is unclear how bacterial colonization interacts with host cells to result in control-like neurobehavioral development. Here, we found variation in the ability of zebrafish-associated microbes to block the hyperactivity phenotype, as measured by distance moved following a light-to-dark transition. Monoassociation with three bacterial isolates resulted in control-like locomotor behavior, two others resulted in an intermediate level of hyperactivity and one failed to block hyperactivity entirely. Association with mixtures of four or five bacterial species resulted in larvae with behavioral phenotypes reflective of those that were conventionalized with a heterogeneous microbiota obtained from the aquaculture facility. In general, these results support those of previous studies in mice and flies in which colonization during development with single strains of bacteria can have different effects on behavior [9, 15]. Monoassociation with an environmentally-associated bacteria did not block the hyperactivity phenotype, but rather these larvae behaved similarly to those in the axenic cohort. This suggests that colonization with microbes that evolved in the host intestinal tract may be required to allow for control-like neurobehavioral development.

We detected two OTUs (OTU6 and OTU8) present in the axenic cohort, as well as a third (OTU7) also present in the monoassociated larvae. These OTUs are in the phylum Bacteriodetes, class Cytophagia, order Cytophagales. Species in this order can be opportunistic and are found mainly in habitats rich in organic material [16]. Cytophagia has previously been detected in the zebrafish intestine [17, 18] and further, was shown to increase in relative abundance in the gut in response to a high fat diet, correlated with markers of inflammation [19]. Because the co-occurrence patterns between OTU6 and OTU8 differed from those of OTU7, we speculate they have different roles. Given that OTU7 co-occurs with the known intestinal bacteria and given its absence in the axenic cohort, we suggest this OTU7 may be a predator in the intestine, as some species within Cytophagales have been identified as facultative predators [20]. They have a gliding motility and biosynthesize metabolites that allow them to extract nutrients by preying on other intestinal bacteria. In contrast, in the axenic larvae, OTU6 and OTU8 were generally the only OTUs detected. These bacteria may be metabolizing organic material produced in the zebrafish gut. Importantly, we did not detect Cytophagales in the larvae with complex microbial communities (CC and AC1). Given that the total read counts were higher in the monoassociated larvae, the lack of detection is unlikely to reflect sequencing limitations. Rather, the complexity and stability of the microbiota within CC and AC1 may serve a protective role and prevent the growth of Cytophagales species. An alternative but not mutually exclusive possibility is that particular species within the communities have adhesion factors that provide protective functions [21].

Given the unexpected presence of the Cytophagales bacteria, we were interested in whether they may have affected larval zebrafish behavior. Using a Bayesian modeling approach, we found a lack of support for the hypothesis that these OTUs affected locomotor behavior in our experiments. Furthermore, the hyperactive behavioral phenotype in the axenic cohort was nearly identical to that observed in our previous studies where the amount of bacteria measured in axenic larvae was significantly lower [4]. The other host-associated strain of bacteria that did not appear to block axenic-related hyperactivity was *A. venetianus*. The role of this bacteria in the zebrafish is unknown. It may be primarily skin-associated, as *A. venetianus* has been identified as a skin-associated microbe in juvenile snook, *Centropomus undecimalis* [22], and as an antifungal skin-associate in the frog, *Espadarana prosoblepon* [23], though other *Acinetobacter* have been detected in the zebrafish gut [24, 25]. In our experiments, larvae monoassociated with *A. venetianus* had the lowest bacterial abundance compared to all other cohorts, and further, *A. venetianus* was the least impacted by competition in the defined mixtures. In addition, there were very few reads for OTU7 (order Cytophagales) in the *A. venetianus*-associated cohort. Assuming this OTU is present as a predator in the intestine, as outlined above, these lines of evidence all support the proposition that *A. venetianus* is primarily inhabiting the skin of larval zebrafish. Lastly, the other bacteria that did not prevent axenic-related hyperactivity was the environmentally-associated *R. makueensis*, originally isolated from ocean water. However, we note that environmental isolates are not necessarily unable to colonize the zebrafish gut [26] and that additional experiments will be needed to demonstrate to what extent colonization may occur. It may also be worthwhile to examine locomotor behavior in multiple cohorts of axenic larvae serially conventionalized with *R. makueensis*, as it has been shown that microbes not previously known to colonize the zebrafish intestine can evolve this ability [26].

In contrast to the species that did not appear to affect behavior, evidence suggests that those that did mediate neurobehavioral development colonize host intestinal tracts. The bacteria species that fully blocked axenic dark-phase hyperactivity included *V. metoecus*, *V. cholerae,* and *A. veronii,* all of which are common inhabitants of the zebrafish gut. The colonization dynamics of both *Vibrio* and *A. veronii* have been well-studied in the zebrafish intestine [27]. Monocolonization with *D. tsuruhatensis* and *C. testosteroni* resulted in an intermediate behavioral phenotype. *D. tsuruhatensis* has been found in the gut of the grass carp, *Ctenopharyngodon idellus* [28], and at the genus level in the zebrafish gut [25]; *C. testosteroni* has previously been identified in the zebrafish intestine [29]. Notably, while colonization of the host intestinal tract may be necessary for neurobehavioral development, critical follow up experiments will be required to determine specific microbially-associated molecules and host responses required to establish the colonized motility phenotype.

In addition to intestinal colonization, there may be a relationship between absolute bacterial abundance and locomotor behavior. We found a weak but significant negative correlation between locomotor behavior and the cell-equivalent bacteria per larvae, whereby larvae with more bacteria show a pattern of reduced hyperactivity. In previous work, axenic zebrafish exposed at 1 day post-fertilization to 100 or 500 cells/mL of *A. veronii* and 5000 or 25,000 cells/mL of *V. cholerae* induced hypoactivity compared to colonized controls [4]. In the current study, exposure to 100 cells/mL of these two bacteria resulted in behavior that did not differ significantly from either colonized control group (CC or AC1), though notably exposure to these two strains did result in the lowest activity of all groups. In our previous study, it is possible that relatively high doses of bacteria added to the aquarium water resulted in a rate of bacterial immigration into the host that exceeded emigration, potentially causing sickness behavior [30].

Host-associated microbiota function through the dynamics of community ecology [31]. When microbe-free zebrafish are inoculated with bacterial isolates, initially there is little resource competition, competitive exclusion, or predation in the intestinal tract, thus bacteria can grow rapidly [27, 32]. In general, the larvae associated with isolates and defined mixtures had a higher abundance of bacteria compared to the larvae with complex communities. As suggested by the presence of Cytophagales, this may reflect a state of dysbiosis. In the defined mixture experiments, we found that the two host-associated isolates that fully blocked axenic-related hyperactivity performed better in the mixing experiments (*A. veronii* and *V. metoecus*). In other words, while the same number of cells were added to flasks containing axenic zebrafish, these two species yielded the highest normalized abundance and therefore the highest growth rates after 10 days. Competition experiments between *A. veronii* and *V. cholerae* have shown intestinal motility can selectively reduce the abundance of *A. veronii* [33]. However, with mixtures of four or five species, higher-order interactions weaken competition and facilitate diversity [34]. While it may be informative to examine longer-term outcomes in the larvae associated with single isolates or mixtures, these data indicate that species that grow rapidly in isolation outcompeted less robust taxa present in the defined mixtures.

## Conclusions

There is now sufficient evidence to suggest that the microbiome modulates the development of brain and behavior across taxa. As such, moving forward it will be critical to consider the microbiome in the design of genetic and toxicity screens for factors that affect zebrafish neurodevelopment. For example, there is a two-way interaction between host microbiota and xenobiotic exposure, whereby chemicals in the environment can modify the composition of the microbiome, and the microbiome (i.e. all of the microorganisms and their encoded genes and associated functions that colonize a host organism), in turn, can bioactivate or inactive xenobiotics [35]. Further, as our data suggest, intramicrobiome interactions are also important to consider. Given that individual bacterial species have different effects on locomotor behavior, these interactions could substantially affect the results of toxicity screens. In particular, there are significant differences between zebrafish laboratory facilities (e.g. strains, water source and treatment methods, and food sources) that harbor the capacity to significantly alter aquaculture microbiota, even between days within the same facility [36]. Thus, consideration should be given not only toward consistently sampling and reporting the bacterial species present in zebrafish experiments using sequencing methods, but also toward accurately measuring their total abundance. While best practices for the determination of zebrafish contamination by culturing flask water have been described [37], our data from the axenic cohort demonstrate that culturing methods and visual inspection of microbial growth can be inadequate. Thus, there is need for standardization in both ontologies and methodological approaches necessary to label an animal “axenic.” In general, it is possible that variation in study results within and across laboratories represents underappreciated manifestations of host-microbial interactions.

## Methods

### Zebrafish husbandry

All experiments involving zebrafish were approved by the Institutional Animal Care and Use Committee at the U.S. EPA National Health and Environmental Effects Research Laboratory and performed in accordance with appropriate guidelines and regulations. A mixed wild type adult zebrafish line (*Danio rerio*) was used for all studies as previously described [36]. Zebrafish adults were housed on a recirculating system with conditioned reverse osmosis water in 6 L tanks at an approximate density of 8 fish/L, maintained on a 14:10 h light:dark cycle at 28.5 °C (pH = 7.5; conductivity = 800 μS), and fed Gemma Micro (Skretting) once daily and shell free E-Z Egg (Brine Shrimp Direct) twice daily Monday-Friday. On weekends, zebrafish were fed both food sources once daily. For spawning, ~ 80 adults were placed in 10 L angled static breeding tanks overnight. The following morning, adults were transferred to new bottom tanks containing treated reverse osmosis water (60 mg/l sodium bicarbonate and 0.4 g/l Crystal Sea Bioassay Formula Marine Mix) and embryos were collected after 45 min.

### Bacterial isolation

On 0 days post fertilization (dpf), conventionally colonized zebrafish embryos were loaded into T25 tissue culture flasks (two flasks, *n* = 15 embryos/flask) containing 10% Hank’s balanced salt solution (HBSS) and incubated at 26 °C. On 1 dpf, an 80% media change was performed, and flasks were housed statically through 6 dpf. From 6 to 9 dpf, media (10% HBSS) was renewed daily and larvae were fed Gemma Micro 75 (Skretting). Ten dpf larvae were anesthetized on ice then homogenized and serial dilutions were prepared (1:10, 1:100, and 1:1000). One hundred uL was cultured on tryptic soy agar (TSA) and Luria-Bertani (LB) plates (*n* = 4). Plates were wrapped in Parafilm and incubated overnight at 26 °C, then colonies were gently spread to new plates and the process was repeated until visually pure colonies were obtained. Isolates were grown in 5 mL LB broth and incubated overnight at 26 °C with shaking. Optical density (OD) readings were obtained using Nanodrop2000c (OD_600_ X 10) to identify growth phases and approximate bacterial concentrations (cells/mL) (https://www.chem.agilent.com/store/biocalculators/calcODBacterial.jsp.). Cultures were stored in glycerol stocks at − 80 °C until use.

### Whole genome sequencing of host-associated bacterial isolates

We conducted whole genome sequencing on the five isolates of unknown species, which revealed their identity as the following: *Acinetobacter venetianus, Vibrio metoecus, Comamonas testosteroni, Delftia tsuruhatensis,* and *Aeromonas veronii.* For detail on genome sequencing, see Keely et al. (in preparation). Briefly, DNA was extracted from bacterial cultures using a ZR-Duet™ DNA/RNA MiniPrep Kit (Zymo Research, #D7003), and samples were stored at − 80 °C. Paired-end indexed libraries were prepared from genomic DNA of each isolate using the Nextera XT Library Prep Kit (Illumina, Inc., San Diego, CA) and Nextera XT Index Kit (Illumina, Inc.) per the manufacturer’s instructions. Libraries were pooled by volume, supplemented with PhiX Control v3 (Illumina, Inc.) to 5% (v/v) and sequenced using the MiSeq System (Illumina, Inc.) with the 500-cycle MiSeq Reagent Kit v2 (Illumina, Inc.) as instructed by the manufacturer. Whole genome sequencing analysis was performed in PATRIC v3.6.2 [38]. Specifically, sequence reads were trimmed and filtered using TrimGalore [39], assembled using SPAdes [40], and classified using MASH [41].

### Axenic derivation and association with isolates

The experimental design is summarized in Fig. 1. Conventionally colonized (CC) larvae were generated by collecting embryos in untreated fish facility water at 0 dpf. Axenic (AX) larvae were derived following a series of treatments with antibiotics, poly (vinylpyrrolidone)-iodine (PVP-I) solution, and bleach, as previously described [35, 42]. Briefly, embryos were resuspended in 0.2 μm filter-sterilized 10% Hanks’ Balanced Salt Solution (FS-10% HBSS) containing antibiotics (amphotericin B (0.25 μg/ml), kanamycin (5 μg/ml), ampicillin (100 μg/ml), and gentamycin (0.05 mg/ml) for 4 h at 26 °C. At 5 h post fertilization, embryos were treated with 0.5% PVP-I (CASRN 25655–41-8) for 2 min and 0.05% bleach for 20 min and sorted into sterile T25 tissue culture flasks (25 mL of 10% HBSS).

As a control for the derivation process, a subset of axenic embryos was conventionalized with 10 ml of filtered (5 μm) aquaculture facility water (containing microbiota) at 1 dpf to generate the axenic colonized on day 1 (AC1) cohort. In addition, to increase the similarity between CC and AC1 cohorts, CC flasks also received 10 ml of aquaculture facility water on day 1. This aquaculture facility water was syringe-filtered using a sterile 5 μm filter to remove debris. In addition, a portion of 5 μm filtered fish facility water was syringe-filtered using a sterile 0.2 μm filter to generate microbe-free fish facility water. At 1 dpf, CC and AC1 flasks received an 80% media change consisting of 10 ml of microbe-containing 5 μm filtered fish facility water and 10 ml of sterile 10% Hanks’ Balanced Salt Solution (HBSS). In comparison, AX flasks received 10 ml of sterile 0.2 μm filtered fish facility water and 10 ml of sterile 10% HBSS. All flasks were housed statically through 6 dpf. From 6 to 9 dpf, all flasks underwent a daily 80% media change and received 75 kilogray gamma-irradiated Gemma Micro 75 at a final concentration of 0.04% (vol/vol) to eliminate microbial contributions from the food source.

To test sterility, at 1 and 10 dpf, two tryptic soy agar (TSA) plates (Sigma, #22091) were inoculated with 10 μl of media from each flask and examined for aerobic and anaerobic growth. For the AX-derived cohort, additional sterility testing for aerobic growth was done at dpf 6, 7, 8, and 9. At 10 dpf, the sterility of media from axenic flasks was further tested by inoculating 100 μL of flask media into tubes of Nutrient Broth (Sigma, #70122), Brain Heart Infusion Broth (Sigma, #53286), or Sabouraud Dextrose Broth (Sigma, #S3306). Plates and tubes were incubated at 26 °C under aerobic and anaerobic conditions for at least 7 days. One AX flask was found to be contaminated and was excluded from the study.

To generate larvae that were colonized with single or mixed bacteria isolates, an additional subset of axenic embryos were exposed to 100 cells/ml of *A. venetianus*, *V. metoecus*, *C. testosteroni*, *D. tsuruhatensis*, *A. veronii*, *V. cholerae* (ZWU0020), or one of two defined mixtures (AC1:DM1 = *A. venetianus* + *V. metoecus* + *C. testosteroni* + *D. tsuruhatensis + A. veronii*; AC1:DM2 = *A. venetianus* + *C. testosteroni* + *D. tsuruhatensis + A. veronii*). Twenty cells/mL per isolate were used for AC1:DM1 and 25 cells/mL per isolate were used for AC1:DM2 such that there were 100 cells/mL total used for all association groups. In a separate experiment following the same derivation methods, axenic larvae were exposed to 100 cells/mL of environmentally-derived *Rheinheimera makueensis* (AC1:*R. makueensis*; ATCC, Manassas, VA; sp. Nov.; Strain KH87; batch # 63381256; originally isolated off-shore from Kaneohe Bay, Oahu Hawaii). We selected *R. makueensis* because Rheinheimera was a genus level taxon present in previous studies [35, 36]. As our strategy to isolate zebrafish-associated microbes did not yield Rheinheimera species, we opted to use a commercially available strain of Rheinheimera to determine whether monoassociation with an environmentally derived strain of Rheinheimera could block axenic hyperactivity.

### Behavior testing

At 10 dpf, larvae were assessed by visual inspection. Larvae that appeared morphologically normal were removed from flasks using a sterile transfer pipet and placed into 48-well plates containing 500 μl of FS-10% HBSS per well. Plates were sealed, wrapped in Parafilm, and placed in the dark in a temperature-controlled behavior testing room at 26 °C, for at least 2 h prior to testing. For testing, microtiter plates were placed on a Noldus tracking apparatus and locomotor activity was recorded. The light program consisted of a 20-min acclimation period in the dark (0 lx) followed by a test period comprised of a 10- or 20-min light period (18 lx) and a 10- or 20-min dark period (0 lx) for host- and environmentally-associated isolate trials, respectively [4]. In larval zebrafish, exposure to dark after a light period results in a characteristic increase in locomotor activity (light-seeking behavior) [43, 44]. A luminescence level of 18 lx for periods of 10 or 20 min follows behavioral protocols used previously [4, 42, 45]. Videos were analyzed using Ethovision software v.12 (Noldus Information Technology) as previously described [46].

### DNA extraction and 16S rRNA gene sequencing

Microbial DNA was isolated by placing 500 μL of microbial growth collected from LB tubes (i.e. isolates used for monocolonizations) into Red RINO lysis tubes (Next Advance, # REDR5). Drawing from the same flasks from which larvae were taken for behavioral testing, we also examined community identities in all host-associated colonization groups. Whole zebrafish larvae at 10 dpf (3–7 biological replicates, 4–8 larvae per replicate, average of 7.75) were loaded into Red RINO tubes with 500 μL of FS-10% HBSS and anesthetized on ice. There were 3 flasks (i.e., biological replicates) used for CC, AC1, AC1:*D. tsuruhatensis*, and AC1:*V. metoecus*; 4 for AC1:*A. venetianus*, AC1:*A. veronii*, AC1:*C. testosteroni*, AC1:*V. cholerae*, AC1:DM1, and AC1:DM2; and 7 flasks for AX-derived larvae. A higher number of AX flasks was used in order to provide a buffer for flask loss due to contamination [4, 35, 36]. We chose to pool individual larvae to capture the heterogeneity inherent in multiple animals from the same flask (i.e. reared in the same environmental conditions), rather than representing a given flask with only a single larva. Following homogenization of microbial or zebrafish samples using a Bullet Blender, DNA was isolated from each sample using a ZR-Duet™ DNA/RNA MiniPrep Kit (Zymo Research, #D7003), and samples were stored at − 80 °C. Total DNA yield was measured using the Qubit dsDNA High Sensitivity Assay Kit (ThermoFisher, #Q32851) and Qubit 2.0 Fluorometer (ThermoFisher). DNA sequencing of the 16S rRNA gene was done as previously described [4, 36, 42]. Briefly, 250 ng of DNA from each sample was added to PCR reactions along with barcoded primers specific for the V4 region of the 16S rRNA gene [47] and amplified with the Roche FastStart High Fidelity PCR System (Sigma-Aldrich, #4738292001). PCR reactions were run for 2 min at 95 °C, followed by 25 cycles of 30 s at 95 °C, 30 s at 55 °Cn and 1 min at 72 °C, with a 10 min final extension at 72 °C. Triplicate reactions were pooled and products were purified and normalized with the SequalPrep Normalization Plate Kit (ThermoFisher, #A1051001). The DNA library was sequenced on the Illumina MiSeq platform (MiSeq Reagent Kit v2; 500 cycles, Illumina, #MS-102-2003). Positive and negative PCR control reactions were run with every 30 samples and sequenced to assess sequencing error and potential PCR contamination. Positive controls consisted of a mixture of equal concentrations of genomic DNA and negative controls consisted of 10 mM Tris-HCl at pH 8.5, as used in the dilution of DNA extracts.

### Analysis of 16S rRNA gene sequences

MiSeq reads were processed using mothur (v1.40) [48], as previously reported [4]. Reads were filtered by Phred quality (Q30 with 50 nucleotide window length) and removed if they failed to form complete contigs. Read-pair contigs were removed if they contained any ambiguous base calls, homopolymers with more than 8 nucleotides, or if they were greater than 275 nucleotides in length. Failure to align to the V4 region of the Silva 16S rRNA gene reference alignment also resulted in removal (v128). The read-pair contigs were denoised using a preclustering algorithm. The UCHIME algorithm of USEARCH software [49] was used to identify and remove chimeras. A Bayesian classifier and the Ribosomal Database Project (RDP, training set version 16) were used to classify the read-pair contigs with a minimum bootstrap of 80% [50]. Read-pair contigs that did not classify at the level of kingdom or that classified as Archaea, Eukaryota, chloroplasts, or mitochondria were removed from further analysis. An operational taxonomic unit (OTU) table was generated with rows and columns representing samples and bacterial taxa counts (binned at 3% dissimilarity) and their taxonomic assignments.

### Droplet digital PCR (ddPCR) of 16S rRNA genes

Quantities of 16S rRNA genes in zebrafish samples and potential PCR inhibition were determined by ddPCR using the BioRad QX200 Droplet Digital PCR System as previously described [4]. The exogenous internal amplification control did not show inhibition during ddPCR. For CC, AC1, and AX, the number of bacteria per larvae was determined using the average number of 4.2 copies of 16S rRNA gene per bacterial genome [51]. For monoassociation and mixture experiments, we used the median number of 16S rRNA genes per genome (see below for details).

### Statistical analyses

Analyses were done in R version 3.6.1. To analyze behavior data across time points, we used a mixed effects model (lme4 package [52]) with ‘distance moved’ as the dependent variable and ‘colonization status’ and ‘time’ as fixed effects and ‘plate’ as a random effect. ‘Colonization status’ consisted of the 11 different treatment groups. Because flask was not a significant factor in any model, we averaged the distance moved within each light and dark period for individual larvae. ‘Mean distance moved’ and ‘bacteria per larvae’ were non-normally distributed (Shapiro Wilk test), therefore, to examine pairwise differences, the Kruskal-Wallis test and Dunn posthoc analyses with the Benjamini-Hochberg method for *p*-value adjustment were used to determine significance. To examine within-group similarity, multivariate homogeneity of group dispersions was calculated (i.e., within-group distance-to-centroid) on a Bray-Curtis distance matrix using the function betadisper from the R package ‘vegan’ [53]. To examine significance between groups, Tukey’s ‘Honest Significant Difference’ method was applied [53]. To visually examine differences in community structure from the 16S rRNA gene sequencing data, we used the R library phyloseq [54]. The stacked bar plot was generated using OTUs that contributed at least 1% to any of the sample totals. Heat trees were created using the R package metacoder [13]. To visualize the concentration of ‘bacteria per larvae’, we used a log10 transformation. Using the transformed data, we conducted a linear regression with ‘mean distance moved.’ In addition, we conducted a linear regression of the log transformed ‘bacteria per larvae’ with a sum of the 16S rRNA gene sequencing read counts. To examine the potential role of the three contaminant OTUs on locomotor behavior, we used Bayesian ANCOVA in JASP version 0.12.2 [55]. We set ‘colonization status’ as the fixed effect and included the three OTUs as separate covariates (Cauchy prior scale parameter for fixed effects = 0.5; Cauchy prior scale parameter for covariates = 0.354). CC and AC1 were excluded. In an additional model, we included the three OTUs in the null and examined support for the alternative hypothesis. To estimate the number of cell-equivalent bacterial species for each corresponding OTU, the relative abundance was normalized using the ddPCR measurements and calibrated using the number of 16S rRNA genes per genome, written as:

$$ {B}_{ij}=\left(\frac{otu_{ij}}{\sum {otu}_{ij}}\right)\left(\frac{ddPCR_j}{16{S}_{ij}{b_{ij}}^{-1}}\right) $$

where B_ij_ is the cell-equivalent bacterial quantity estimated by using the relative abundance of the ith otu divided by the total number of otu reads in sample j. The ddPCR quantity of 16S rRNA gene at 10 dpf (ddPCR_ij_) was calibrated by the number of 16S rRNA genes (16S_ij_) per identified species (b_ij_). Data on the median number of 16S rRNA genes per genome was obtained from Espejo and Plaza [56].

## Supplementary Information

**Additional file 1: Fig. S1**. Bacterial abundance (log_10_) measured by ddPCR and total reads (log_10_) measured by 16S rRNA gene sequencing show a positive linear relationship (*p* = 2.8 × 10^− 10^, *R*^*2*^ = 0.62). **Table S1**. Generation, generation time, and growth rate for monoassociation and mixture experiments.

**Additional file 2:** Data files and R script used to generate figures and conduct statistical analyses.

## Data Availability

All data generated during this study are included in this published article and its supplementary information files. In addition, the datasets are available by searching for the manuscript title at https://catalog.data.gov.

## Abbreviations

AC1: Axenic colonized on day 1
AX: Axenic
CC: Conventionally colonized
AC1:DM1: Defined mixture 1, containing *A. venetianus* + *V. metoecus* + *C. testosteroni* + *D. tsuruhatensis* + *A. veronii*
AC1:DM2: Defined mixture 2, containing *A. venetianus* + *C. testosteroni* + *D. tsuruhatensis* + *A. veronii*
dpf: Days post-fertilization
