# Supplementary material for: Monoassociation with bacterial isolates reveals the role of colonization, community complexity and abundance on locomotor behavior in larval zebrafish
Source: Anim Microbiome. 2021 Jan 21;3:12. doi: 10.1186/s42523-020-00069-x (PMC7818562; doi:10.1186/s42523-020-00069-x)
Supplement: Supplementary file 1 — Additional file 1: Fig. S1. Bacterial abundance (log10) measured by ddPCR and total reads (log10) measured by 16S rRNA gene sequencing show a positive linear relationship (p = 2.8 × 10− 10, R2 = 0.62). Table S1. Generation, generation time, and growth rate for monoassociation and mixture experiments. [file 42523_2020_69_MOESM1_ESM.docx]

**Figure S1.** Bacterial abundance (log_10_) measured by ddPCR and total reads (log_10_) measured by 16S rRNA gene sequencing show a positive linear relationship (p = 2.8x10^-10^, R^2^ = 0.62).

**Table S1**. Generation, generation time, and growth rate for monoassociation and mixture experiments.

| **Species** | **Generations ± SD** | **Generation time (hours) ± SD** | **Growth rate ± SD** |
| --- | --- | --- | --- |
| *V. metoecus* | 9.91 ± 0.79 | 21.89 ± 1.83 | 0.0318 ± 0.0025 |
| *V. cholerae* | 11.14 ± 0.84 | 19.47 ± 1.50 | 0.0357 ± 0.0027 |
| *A. veronii* | 8.74 ± 0.30 | 18.86 ± 0.99 | 0.0369 ± 0.0019 |
| *C. testosteroni* | 11.85 ± 0.53 | 18.26 ± 0.78 | 0.0380 ± 0.0017 |
| *A. venetianus* | 11.48 ± 0.60 | 24.74 ± 0.84 | 0.0280 ± 0.0010 |
| *D. tsuruhatensis* | 10.84 ± 0.31 | 19.94 ± 0.59 | 0.0348 ± 0.0010 |
| DM1 | 11.11 ± 0.21 | 19.45 ± 0.38 | 0.0357 ± 0.0007 |
| DM2 | 11.45 ± 0.84 | 18.93 ± 1.31 | 0.0368 ± 0.0027 |

The number of generations g_j_ for exponential growth [1] was calculated using:

$$g_{j}=\frac{\ln\left( \frac{{ddPCR}_{j}}{{16S}_{j}{b_{j}}^{-1}} \right)-ln(n_{1j})}{ln(2)}$$

where n_1j_ is the average quantity per larva of inoculated cells for sample j at 1 dpf. The number or the average of 16S rRNA genes per species were respectively used for monoassociation and mixture experiments. The mean generation time was calculated by dividing the total hours of growth by g_j_. Finally, the growth rate was calculated as follows:

$$u_{j}=\frac{ln\left( \frac{{ddPCR}_{j}}{{16S}_{j}{b_{j}}^{-1}} \right)-ln(n_{1}j)}{t}$$

where *t* is the amount of elapsed time (216 hours) of the experiment (i.e., the number of hours from conventionalization at 24 hpf and analysis at 240 hpf).

References

1. Maier RM, Pepper IL: Chapter 3 - Bacterial Growth**.** In *Environmental Microbiology.* Third edition. Edited by Pepper IL, Gerba CP, Gentry TJ: Academic Press; 2015: 37-56
